# Epidural varicosis as a possible cause of radicular pain: a case report

**DOI:** 10.1186/1752-1947-5-537

**Published:** 2011-11-01

**Authors:** Stefan Endres

**Affiliations:** 1 Orthopädie und Unfallchirurgie Elisabeth-Klinik Bigge/Olsberg, Heinrich-Sommer-Strasse 4, 59939 Olsberg, Germany

## Abstract

**Introduction:**

The incidence rate of epidural varicosis has declined by 0.07% to 1.2% since the introduction of computed tomography and magnetic resonance imaging. Despite the use of these modern imaging methods it can still be difficult to distinguish the diagnosis of epidural varicosis from other causes, such as nucleus pulposus prolapse.

**Case presentation:**

We present the case of a 48-year-old Caucasian woman who had been experiencing sciatic pain for seven years. A physical examination showed nerve root pain at L5 on the right side, with positive signs of neurotension. During an elective hysterectomy due to endometriosis, unusually pronounced varicosis in her lesser pelvis was seen that had not previously been detected. Postoperatively, our patient developed a symptomatic pulmonary embolism. Findings from magnetic resonance tomography of her lumbar spine, in conjunction with our patient's history, were considered by the radiologist to be indicative of epidural varicosis. No further pathological abnormalities that could have been the cause of the nerve root pain were found.

**Conclusions:**

In cases of epidural varicosis with irritation of neural structures as a result of inferior vena cava hypoplasia, surgical treatment leads to unsatisfactory results. Significantly better results can be achieved by resolving the cause of the vena cava pathology. In cases of hypoplasia or aplasia of the inferior vena cava this is not always possible; consequently, as in the case of our patient, only a symptomatic therapy in combination with an anticoagulant and compression therapy can be performed.

## Introduction

Low back pain with unilateral or bilateral radicular pain is mainly caused by protrusions of the intervertebral disc tissue that come into contact with the spinal nerves. Sometimes neurological deficiencies, in the form of paresis or bladder and rectal dysfunction, may also occur. The diagnosis in most cases can be made via computed tomography (CT) or magnetic resonance imaging (MRI).

The impingement on nervous tissue by spinal epidural varices has only rarely been described in the literature [1-4]. Despite the use of modern imaging methods (such as MRI, myelography and CT), it can still often be difficult to distinguish the diagnosis of epidural varicosis from other causes. Epidural varicosis often masquerades as a herniated nucleus pulposus, and the definitive diagnosis is usually made on operation.

We present the case of a 48-year-old Caucasian woman who was treated under a tentative diagnosis of a multisegmental lumbar disc protrusion for some years. After a diagnosis of inferior vena cava hypoplasia and updated diagnostic imaging, a diagnosis of epidural varicosis was finally made. The diagnosis, pathophysiology and treatment of this condition are discussed.

## Case presentation

We present the case of a 48-year-old Caucasian woman who had been experiencing sciatic pain for seven years. Her symptoms varied in intensity, and intermittent ambulant medical treatment was administered. When her symptoms increased, with the onset of sciatica radiating from the fifth lumbar nerve root on the right side, an MRI scan of her spine was performed and an intensification of conservative therapeutic methods under stationary conditions was planned. The MRI results (0.5T) were interpreted as a prolapse of the L4/L5 lumbar intervertebral disc, abutting the L5 thecal sac and nerve root, causing the pain in her leg.

Conservative treatment with a series of periradicular infiltrations (including bupivacaine and triamcinolone) in combination with physical therapy resulted in a decrease in her symptoms and our patient was discharged. Subsequently, she underwent an elective hysterectomy due to endometriosis. During this surgery, unusually pronounced varicosis was found in her lesser pelvis that had not previously been detected. Postoperatively, our patient developed a symptomatic pulmonary embolism. Consequently, further evaluation of adjacent structures and diagnostic tests for thrombophilia were initiated. The pulmonary embolism was found to be caused by hypoplasia of her inferior vena cava, with a bilateral occlusion of her vena iliaca communis. A diagnostic evaluation showed that a collateral pathway with ectatic enlargement of the veins of her lesser pelvis had also developed. Anticoagulant medication in combination with compression therapy was recommended because a surgical correction of this malformation was not possible.

Subsequently, our patient was again admitted to our hospital because of an exacerbation of the nerve root irritation. She had classic root tension signs (straight leg raise and bow string tests). In addition, a greater level of pain was experienced with increased intra-abdominal pressure (when, for example, coughing, sneezing or pushing). More severe neurological deficiencies, in the form of paresis or bladder and rectal dysfunction, were not found. She was by this time severely incapacitated and bedridden.

**Figure 1:**
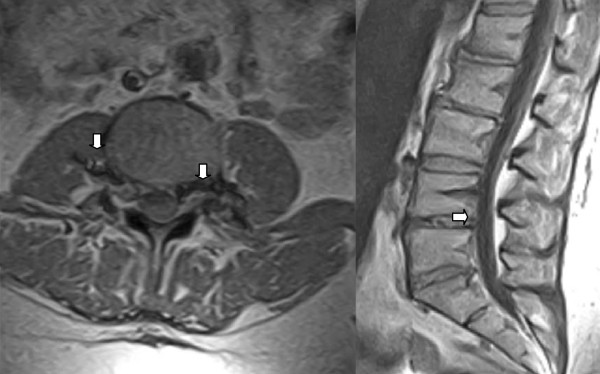
**Epidural varicosis (arrows)**. MRI scan of the lumbar portion (transversal and sagittal).

Bearing in mind the hypoplasia of her inferior vena cava, a repeat MRI scan (1.5T) was performed. The MRI results, in conjunction with our patient's history, were considered by the radiologist to be indicative of epidural varicosis. No further pathological abnormalities that could cause the nerve root pain were found (Figure 1).

According to our vascular surgeons, no surgical correction for the hypoplasia of her inferior vena cava was possible because it was a congenital defect. The optimal therapy to manage the progressive pain symptoms of our patient was then considered. Due to the increased risk of bleeding, the consideration of surgical intervention was abandoned and she was treated with peripheral analgesics in combination with low-dose pregabalin, with satisfactory results. In addition, compression therapy (class II) combined with Marcoumar (phenprocoumon) was carried out, which led to an acceptable decrease in her symptoms (target international normalized ratio; 2.0 to 3.0).

To date, our patient still complains of sciatic pain on her right side, but is able to work while on intermittent pain medication.

## Discussion

MRI is an important tool in the diagnosis of radicular complaints. A review of the recent literature and the case of our patient shows that the presence of epidural varicosis, without also being aware of a vascular abnormality, can easily be misinterpreted as being herniated disc tissue [5]. Thrombosed veins appear hyperintense on T1-weighted and T2-weighted images. Depending on the degree of thrombosis, an epidural vein on T2-weighted images may appear hypodense and hyperintense. Therefore epidural varicosis is often misinterpreted as herniated lumbar discs [6,7].

In the literature, several pathophysiological models for the formation of venous epidural vascular anomalies are discussed. Gümbel *et al*. postulated the possibility of primary epidural varicose veins without any underlying or extra intraspinal pathology [8]. Wong *et al*. suggested that varicose veins are due to the epidural mechanical compression of the venous plexus by disc herniations, spondylolisthesis or spinal stenosis [9]. Through venous stasis, an epidural vein thrombosis may occur over time with subsequent irritation of nerve structures.

Epidural varicosis as a result of an obstruction of the inferior vena cava has frequently been described in the literature. When an obstruction and/or occlusion of the inferior vena cava and vena iliaca communis is present, there is increased blood flow into the azygos and hemiazygos veins. Expansion of the epidural venous plexus, with potential compression of the neuronal structures, also occurs. Treatment with anticoagulant medication in combination with compression therapy, as in the case of our patient, is usually sufficient [10].

In the literature, the alternative possibilities of thrombolysis and surgical intervention have been described. However, the results of thrombolysis are not convincing, so it is rarely used [10]. Genevay *et al*. [1] consider that surgical treatment of an epidural varix is obligatory, but only if a neurological symptom is present. With respect to the nature of the surgery, different approaches exist. Reports on surgical thermocoagulation of the venous plexus [2,9-12], interventional techniques [13] or surgical compression of the venous plexus with a resorbable gelatin sponge [14] have been reported. In most cases, this leads to a good surgical result with significant reduction of the symptoms [9]. In cases of severe epidural varicosis due to a faulty inferior vena cava and dilation of all lumbar veins, the advice is against surgical intervention. This is based on unsatisfactory surgical results [14] and disproportional surgical risk [12].

## Conclusions

Epidural varicosis with irritation of nerve structures observed on MRI should direct attention to the possibility of an inferior vena cava thrombosis or compression. In such cases, an MRI scan of the region around the inferior vena cava should be performed.

It is proposed that epidural varicosis due to inferior vena cava pathology can cause radicular pain. Knowledge of the existence of such a condition and its possible etiologies may assist in its recognition and improve clinical management of affected patients.

In cases of epidural varicosis with irritation of neuronal structures that develop due to hypoplasia of the inferior vena cava, surgical intervention gives unsatisfactory results [9]. In contrast, interventions that resolve the cause of the pathology in the inferior vena cava lead to significantly better results [11].

This is not always possible where there is hypoplasia and/or aplasia of the inferior vena cava, so, as in our patient's case, only symptomatic therapy in combination with anticoagulation and compression therapy is possible.

## Consent

Written informed consent was obtained from the patient for publication of this case report and any accompanying images. A copy of the written consent is available for review by the Editor-in-Chief of this journal.

## Competing interests

The author declares that they have no competing interests.
